# Investigating *Klebsiella pneumoniae* biofilm preservation for scanning electron microscopy

**DOI:** 10.1099/acmi.0.000470.v3

**Published:** 2023-02-03

**Authors:** Renee M. Fleeman, Michelle Mikesh, Bryan W. Davies

**Affiliations:** ^1^​ Department of Molecular Biosciences, The University of Texas at Austin, Austin, TX 78712, USA; ^2^​ Center for Biomedical Research Support, The University of Texas at Austin, Austin, TX 78712, USA; ^3^​ Center for Systems and Synthetic Biology, The University of Texas at Austin, Austin, TX 78712, USA; ^4^​ John Ring LaMontagne Center for Infectious Diseases, The University of Texas at Austin, Austin, TX 78712, USA; ^†^​ Present address: Division of Immunity and Pathogenesis, Burnett School of Biomedical Sciences, College of Medicine, University of Central Florida, Orlando, FL 32837, USA

**Keywords:** biofilm, *Klebsiella pneumoniae*, scanning electron microscopy

## Abstract

*
Klebsiella pneumoniae* biofilm formation is associated with chronic and relapsing infections. Scanning electron microscopy (SEM) is a powerful tool for characterizing biofilm structure and studying their formation. Reliable visualization of biofilm structure requires careful sample preservation, otherwise there may be loss of non-covalent interactions that are susceptible to damage during the dehydration and washing preparation steps. However, no standard procedure has been adopted in the literature to fix *K. pneumoniae* biofilm for scanning electron microscopy studies. This lack of standardization makes it challenging to compare results between studies and determine the degree to which native structures have been preserved. To advance this critical area of study, we investigated different scanning electron microscopy fixation methods for *K. pneumoniae* biofilm preservation. Our study reveals the impact preparation steps can have on retaining in biofilm architecture observed using scanning electron microscopy. Using fixation methods developed through our studies, we show that although species that overproduce capsular extracellular polysaccharides produced more robust biofilms, *K. pneumoniae* can form a developed biofilm in the absence of capsular polysaccharides.

## Data Summary

There is no supporting external data generated for this work.

## Impact Statement

*Klebsiella pneumoniae* is an increasing threat to human health due to extreme drug resistance. Biofilms formed by *K. pneumoniae* aid in evasion of the immune system and antibiotic therapy resulting in chronic and relapsing infections. Scanning electron microscopy (SEM) is a powerful tool to observe the structure and topography of *K. pneumoniae* biofilms. Preservation of the components of biofilms is critical to inform our understanding of biofilm formation and its native structure. There is no standard for fixation of *K. pneumoniae* biofilm for scanning electron microscopy visualization and different techniques are used with each study. Here we describe our investigation of factors that influence retention of biofilm structure for SEM and demonstrate how biofilm structure can be lost with improper fixation. Using a fixation method developed during our investigation we show that extracellular polysaccharide capsule is not required to form complex biofilm structures. Our results reveal the importance of proper fixation when preserving *K. pneumoniae* biofilms for SEM and its impact on observing difference in biofilm structure between strains.

## Introduction

*Klebsiella pneumoniae* is an opportunistic pathogen that has gained notoriety in recent years due to its rapid increase in drug resistance [1–3]. *K. pneumoniae* biofilm formation increases resistance to antibiotics and the host immune system leading to chronic infections with frequent relapsing [4, 5]. Characterization of biofilms using scanning electron microscopy (SEM) allows for a detailed view and understanding of these complicated heterogenous structures in various species [6–10]. However, there are not standardized procedures to preserve *K. pneumoniae* biofilm when preparing for SEM imaging, which has led to different methods being used throughout the literature [7–9, 11, 12]. Understanding how different fixation methods influence retention of biofilm components is important for interpreting SEM biofilm studies.

Biofilms are heterogenous structures with chemical and cellular variation throughout the matrix [13]. These structures are composed of many biomolecules that have complex interactions; therefore, its structure should be treated like tissues when preparing fixative to preserve the structures before dehydration of the sample for SEM [14–17]. Improving biofilm preparation for SEM has shown promise for Gram-positive *Streptococcus mutans *, *Staphylococcus aureus *, *Scardovia wiggsiae* and the Gram-negative *Mannheimia haemolytica* [6, 18]. The fixation step in biofilm preparation for SEM visualization necessitates preservation of the non-covalent interactions that are susceptible to damage during the dehydration and washing steps [16]. Therefore, our focus is to increase preservation of these delicate interactions that hold *K. pneumoniae* biofilms together and prevent their collapse [19].

Our previous work investigating host defense peptides necessitated robust preservation of the biofilm matrix for SEM imaging to show the effects of peptide biofilm disruption [20]. Here we describe our investigation of SEM fixation methods for *K. pneumoniae *. We tested the aldehyde component of the fixative to identify the benefit of adding paraformaldehyde to the glutaraldehyde fixative because this combination has shown to be a superior aldehyde fixative of proteins for SEM [6, 18]. We assessed the effects of adding tannic acid to this combination because it has shown importance in preserving lipids for SEM [21, 22]. This is not commonly used when assessing *K. pneumoniae* biofilms but has been used previously to optimize visualization of the biofilm of *Sc. wiggsiae* and *St. mutans* [6]. Cationic dyes are used to preserve polysaccharides for SEM because they form ionic bonds with anionic polysaccharides [23]. We tested both ruthenium red and alcian blue in our fixations to determine variations in the resulting preservation. From our studies, we developed an improved fixation procedure that retained robust biofilm architecture and used it to observe biofilms from hypermucoviscous *K. pneumoniae *, non-hypermucoviscous *K. pneumoniae *, and isogenic mutants lacking an extracellular polysaccharide capsule. Our results reveal the importance of SEM preparation when preserving *K. pneumoniae* biofilms and reveal that extracellular polysaccharide capsule is not required to form complex biofilm structures.

## Methods

### Bacterial isolates and growth conditions

Hypermucoviscous *K. pneumoniae* NTUH K2044 was used for the method optimization in this study [24]. The SEM characterization of parental and isogenic mutants utilized *K. pneumoniae* KPPR1S, a K2 serotype hypermucoviscous isolate as the parental strain and an isogenic mutant of this isolate Δ*wcaJ,* devoid of an extracellular capsule [25]. *K. pneumoniae* MKP103 from the Maniol lab was also used next to its capsule transposon mutant *wza::180 T_30_* [26].The bacterial cultures were grown overnight in LB media at 37 °C with shaking at 225 r.p.m. The biofilms were seeded by inoculating 1 ml using biofilm media (tryptic soy broth, 3 % NaCl and 0.5 % glucose) with the respective *K. pneumoniae* isolates at an OD_600_ of 0.5 to a 35×10 mm petri dish containing a square of ACLAR® (Ted Pella) film, sealed with parafilm, and growing the culture for 24 h at 37 °C without shaking. ACLAR® is transparent, chemically inert material that is stable for use in scanning electron microscopy [27].

### Scanning electron microscopy

The biofilms on ACLAR® film (Ted Pella Cat. No. 10501–10) were removed from the petri dish they were grown in. ACLAR® discs were fixed overnight with the different fixation recipes listed below (Table 1). All reagents used for sample fixation were electron microscopy grade reagents. Briefly, our basic fixative solution was prepared in 0.2M Na Cacodylate buffer pH 7.4 to achieve 4 % glutaraldehyde (Electron Microscopy Sciences, Aqueous Glutaraldehyde EM Grade 25 and 50 %; Cat. No. 16 220 and 16320) with 0.15 % Ruthenium Red (RR) in a final concentration of 0.1M buffer, followed by 0.15 % RR in 1 % osmium tetroxide. Our second method included 4 % glutaraldehyde and 2 % paraformaldehyde (Electron Microscopy Sciences, paraformaldehyde 20% Aqueous EM Grade; Cat. No. 15713) with 0.15 % RR in 0.1M Na Cacodylate buffer, pH 7.4, followed by 0.15 % RR in osmium tetroxide. Given that biofilm is very ‘tissue-like’ in nature, composed of multiple components with structural elements, we used a paraformaldehyde fixative mix I generally used on tissue, or a modified Karnovsky’s type [28]. Our third method was 4 % glutaraldehyde, 2 % paraformaldehyde and 1 % tannic acid with 0.15 % RR in buffer, followed by RR in osmium tetroxide. The tannic acid addition was tried either in the fixation, or in combination with osmium tetroxide after washing off the fix for both RR and alcian blue (AB) (Sigma-Aldrich Cat. No. A5268-10G) dyes. For the trials using AB in place of ruthenium red, 0.15 % AB was added to the fixation, then stained with 1 % osmium tetroxide with 0.15 % AB in 0.1M Na Cacodylate buffer, pH 7.4.

**Table 1.** Fixation protocol variations*

Step	Basic fixation	Aldehyde improved	Improved fixation
**Fixation**	4 % Glutaraldehyde	4 % Glutaraldehyde	4 % Glutaraldehyde
	--	2 % paraformaldehyde	2 % paraformaldehyde
	0.15 % RR	0.15 % RR or AB	0.15 % RR or AB
	0.1M Na Cacodylate buffer pH 7.4	0.1M Na Cacodylate buffer pH 7.4	0.1M Na Cacodylate buffer pH 7.4
	--	--	1 % tannic acid
**Washing**	Na Cacodylate buffer pH 7.4	Na Cacodylate buffer pH 7.4	Na Cacodylate buffer pH 7.4
**Post Fixation**	0.15 % RR	0.15 % RR or AB	0.15 % RR or AB
	1 % osmium tetroxide	1 % osmium tetroxide	1 % osmium tetroxide
**Dehydration**	Graded alcohols†	Graded alcohols†	Graded alcohols†
**Drying**	Graded HMDS‡	Graded HMDS‡	Graded HMDS‡
**Sputter coating**	12 nm platinum	12 nm platinum	12 nm platinum

*Abbreviations used: ruthenium red (RR), alcian blue (AB) and hexamethyldisilazane (HMDS).

†Graded alcohols: 50 %, 70 %, 90 %, 95 % and finally absolute EtOH (on molecular sieves).

‡Drying ratios: 50 %:50 % absolute EtOH : HMDS, followed by 100 % HMDS.

Fixed cells were then gently washed in buffer with cationic dye, then stained with 1 % osmium tetroxide with cationic dyes and with or without tannic acid as appropriate in buffer for approximately 2 h. The samples were washed gently with water, then dehydrated through graded alcohols (Table 1), transferred to 1 : 1 absolute ethanol in hexamethyldisilazane (HDMS) for 10 min, then 5 min in HDMS, and air dried for 30 min. Samples were sputter coated with 12 nm platinum: palladium (Pt: Pd) and imaged with an SE2 detector in a Zeiss Supra SEM under high vacuum conditions with 5 kV accelerating voltage. All experiments were performed in biological triplicate. The figure images are representative of results for each test condition.

## Results

### A combination aldehyde fixative is superior to glutaraldehyde alone

To assess individual components for SEM preparation and their effects on the resulting image we started by investigating the aldehyde component of the fixation. Our basic fixation method includes glutaraldehyde, the standard aldehyde fixative to preserve proteins for SEM, combined with ruthenium red, a cationic dye to preserve the polysaccharides (Fig. S1A, available in the online version of this article). Although not always used when visualizing biofilm, cationic dyes are necessary for polysaccharide preservation and ruthenium red has been shown to preserve capsular polysaccharides for SEM [6].

**Fig. 1.**
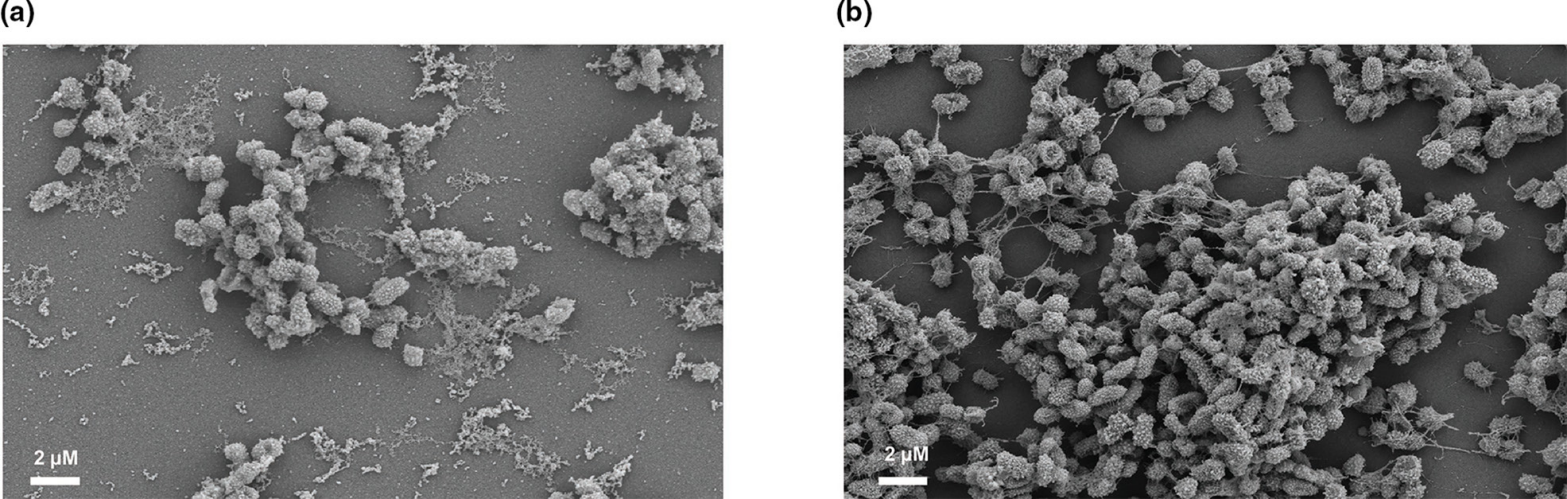
A combination aldehyde fixative solution is superior to glutaraldehyde alone. The figure shows fixation of *K. pneumoniae* NTUH K2044 with glutaraldehyde and ruthenium red (Fig. 1a) or fixation with a combination of glutaraldehyde, paraformaldehyde, and ruthenium red (Fig. 1b). All figures show SEM images with 4000x magnification.

We first tested the effects of adding paraformaldehyde to the glutaraldehyde fixation step. Paraformaldehyde forms methylene bridges resulting from cross-linking of its -CH2 groups [29], while glutaraldehyde forms cross-linking of proteins using -CHO groups [30]. As shown in tissue preparation, paraformaldehyde quickly penetrates the sample resulting in preservation of the overall tissue structure, while glutaraldehyde allows for strengthening of this structure by obtaining more dense cross-linking because it has two -CHO groups per molecule [31]. Furthermore, this combination of aldehydes has been shown to increase preservation of structures within the biofilms of Gram-positive species [6, 18].

We chose to use *K. pneumoniae* NTUH K2044 to test our SEM preparation because of the clinical relevance of this isolate [24]. This isolate is a hypervirulent species known to cause devastating infections leading to liver abscesses and has a hypermucoviscous phenotype provided by an increase in polysaccharide capsule surrounding the cell [32]. We found that although the combination of glutaraldehyde and paraformaldehyde appeared to preserve more biofilm associated cells then glutaraldehyde alone (Fig. 1). Samples fixed with only gluteraldehyde presented sparse and seperated cells with intercellular connections stretching to maintain connections between bacteria (Fig. 1a). In contrast, using both gluteraldehyde and paraformaldehyde more cells were presevered and they were densely packed creating elevation to the biofilm (Fig. 1b). While these images do not allow a quantiative evaluation of polysaccharide, they do suggest that the combination of glutaraldehyde and paraformaldehyde promotes better retention of *K. pneumoniae* biofilm associated cellular density than glutaraldehyde alone.

### Biofilm preservation varies with cationic dyes

Cationic dyes have been used to preserve polysaccharides in SEM preparation and are a necessary component to the fixation step. We used ruthenium red for fixation of the biofilm for Fig. 1 as it is a cationic dye previously shown to preserve the biofilm components [6]. However, alcian blue has been shown to have a role in preserving a variety of polysaccharides for electron microscopy [33] (Fig. S1B). This cationic dye has a phthalocyanine core with four isothiouronium side chains allowing for bulkiness in addition to charge, while ruthenium red has a more linear structure in comparison [34]. We hypothesized the different structures of these dyes would lead to variations in biofilm preservation.

**Fig. 2.**
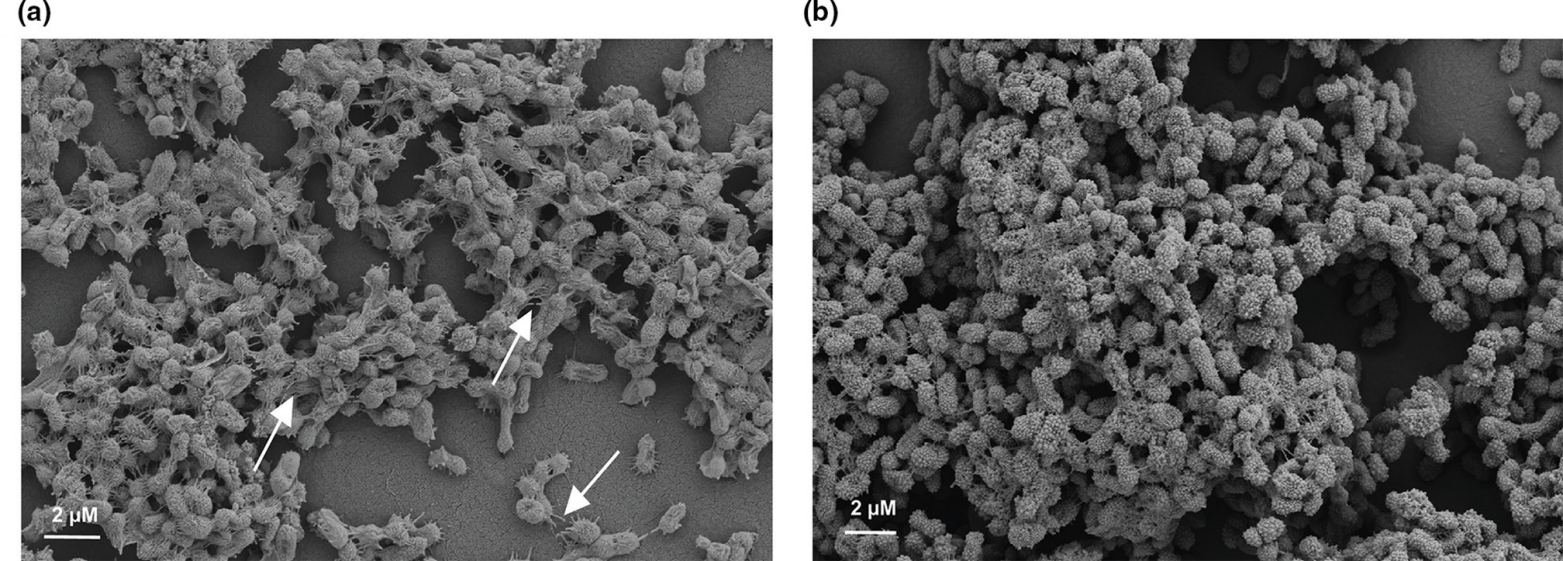
Cationic dyes with different structures do not similarly preserve the biofilm matrix. The figure shows *K. pneumoniae* NTUH K2044 SEM following fixation with alcian blue (Fig. 2a) or ruthenium red (Fig. 2b). White arrows highlight surface associated matrix material. All figures show SEM images with 4000x magnification.

Using the combination glutaraldehyde with paraformaldehyde fixation from Fig. 1b, we found that both cationic dyes led to similar preservation of the *K. pneumoniae* NTUH K2044 biofilm matrix and cellular density (Fig. 2a, b). The ruthenium red fixative (Fig. 2b) combination allowed for preservation of an abundance of biofilm matrix coating the *K. pneumoniae* cells, with little surface associated matrix attaching to the ACLAR® support. Biofilm matrix surrounding *K. pneumoniae* appeared slightly less dense using alcian blue (Fig. 2a) but there was an abundance of ACLAR® support associated matrix preserved (white arrows).

### The addition of tannic acid increases visualization of intercellular connections

We next wanted to assess the addition of tannic acid to the fixative. Tannic acid is a mix of polyglycol anions that form complexes between proteins and lipids or carbohydrates to decrease damage from SEM preparation [16, 21]. With the importance of non-covalent interactions in the biofilm matrix, we chose to assess the addition of tannic acid to the fixation step. To identify how the effects of tannic acid vary based on the cationic dye used during preparation, we tested tannic acid in combination with both ruthenium red and alcian blue. We continued to use the combination of paraformaldehyde and glutaraldehyde in the fixation with both cationic dyes but varied when the tannic acid was added to the preparation.

**Fig. 3.**
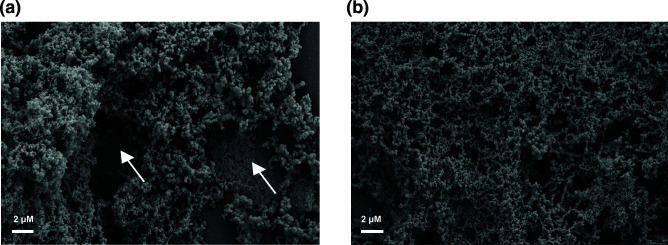
Increased matrix preservation with tannic acid. The figure shows *K. pneumoniae* NTUH K2044 SEM following fixation with alcian blue with tannic acid (Fig. 3a) and ruthenium red with tannic acid (Fig. 3b). White arrows show gaps in the biofilm matrix. All figures show SEM images with 4000x magnification.

Interestingly, the addition of tannic acid to the fixative overall appeared to preserve more biofilm material compared to cationic dyes with glutaraldehyde and paraformaldehyde alone (Fig. 2). Furthermore, *K. pneumoniae* NTUH K2044 biofilm preservation appeared better with ruthenium red with tannic acid (Fig. 3b) compared to with alcian blue dye combined with tannic acid (Fig. 3a) This was evident by visualization of more individual cells exposed and gaps present (white arrows) in the biofilm matrix when alcian blue is in the fixative (Fig. 3a). The gaps within the biofilm matrix were smaller when ruthenium red was in the fixative and individual cells were deeply buried within the matrix (Fig. 3b). These results highlight the importance of tannic acid in addition to the cationic dyes for preservation of *K. pneumoniae* biofilm matrix and that ruthenium red afforded slightly superior preservation with this fixation step.

**Fig. 4.**
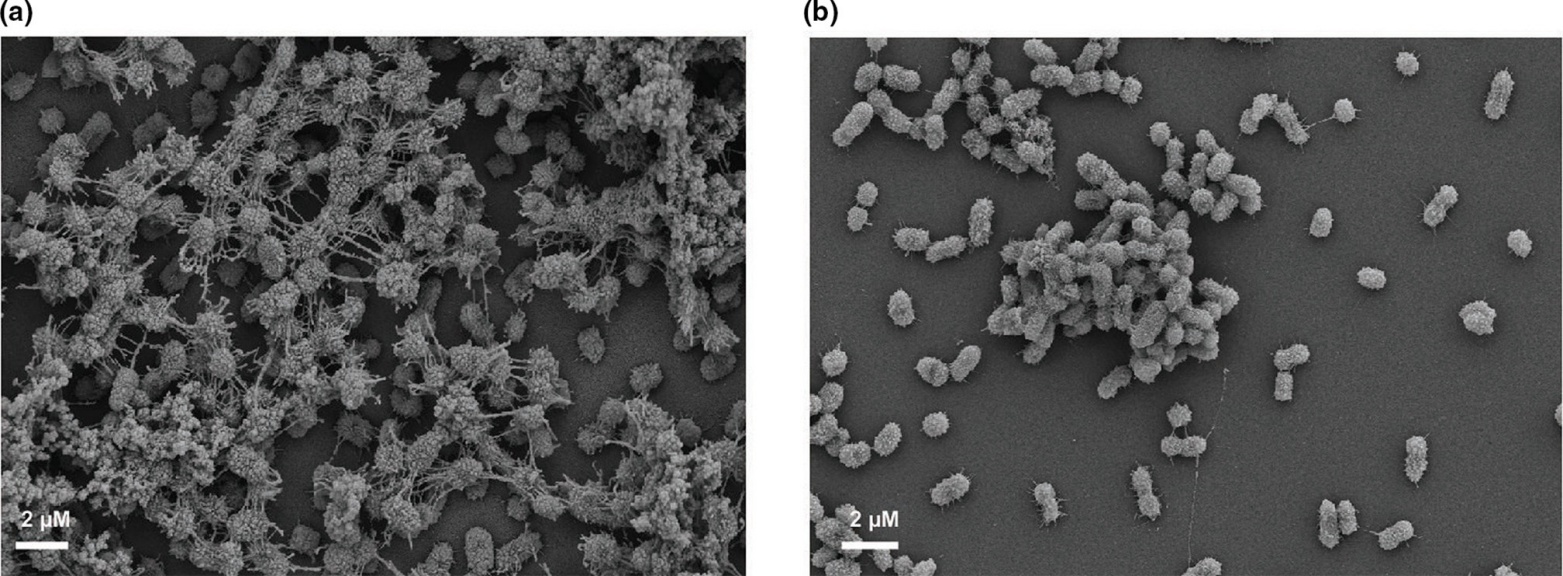
Tannic acid treatment following fixation with cationic dyes. The figure shows *K. pneumoniae* NTUH K2044 SEM with tannic acid treatment following either alcian blue (Fig. 4a) or ruthenium red fixation (Fig. 4B). All figures show SEM images with 4000xmagnification.

The effect of tannic acid has been shown to vary depending on if it is added to the primary fixative or added subsequently to primary fixation [6, 21]. To determine if treatment with tannic acid after fixation would have a different effect on the biofilm preservation, we prepared samples with the tannic acid added after the initial fixation with the cationic dyes. With this preparation we saw a profound difference based on the cationic dye the tannic acid followed. Specifically, tannic acid treatment following fixation with alcian blue resulted in a circular pattern of matrix around the cells (Fig. 4a). This could be from the channels present in the biofilm becoming more apparent with this treatment or it could merely be an artifact of the tannic acid reacting with the alcian blue fixative. The tannic acid treatment when following ruthenium red fixation had a devastating effect on the biofilm preservation, as the fixed biofilm matrix was washed away leaving attached cells absent of matrix material (Fig. 4b). We concluded that the inclusion of tannic acid is important for biofilm preservation when added to the fixation step, but caution should be taken when added post-fixation.

### Improved sample preparation reveals strain biofilm variations

**Fig. 5.**
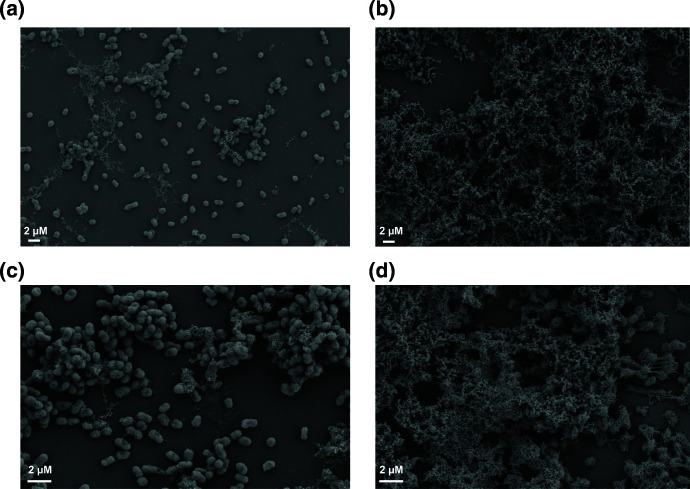
Optimal preservation of matrix material with improved fixation. The figures show *K. pneumoniae* NTUH K2044 biofilm fixed with the basic fixation (Fig. 5a) or with the improved fixation (Fig. 5b) at 2000x magnification. Fig. 5c, d are basic fixation and improved fixation, respectively, at 4000x magnification.

Based on the conditions tested here, the best fixation method to retain biofilm architecture included paraformaldehyde, glutaraldehyde, tannic acid and ruthenium red (Fig. 3b). This preserved the most cell associated matrix that encases the biofilm associated cells (Fig. 5). With the basic fixation (glutaraldehyde and ruthenium red) (Table 1), *K. pneumoniae* NTUH K2044 cells were visible and little matrix was observed (Fig. 5a). However, with the improved fixation method (Table 1) cells were covered in a robust matrix (Fig. 5b). At higher magnification samples stained with the basic fixation method (Fig. 5c) have minimal matrix material and intracellular connections compared to cells stained with the improved fixation method (Fig. 5d).

**Fig. 6.**
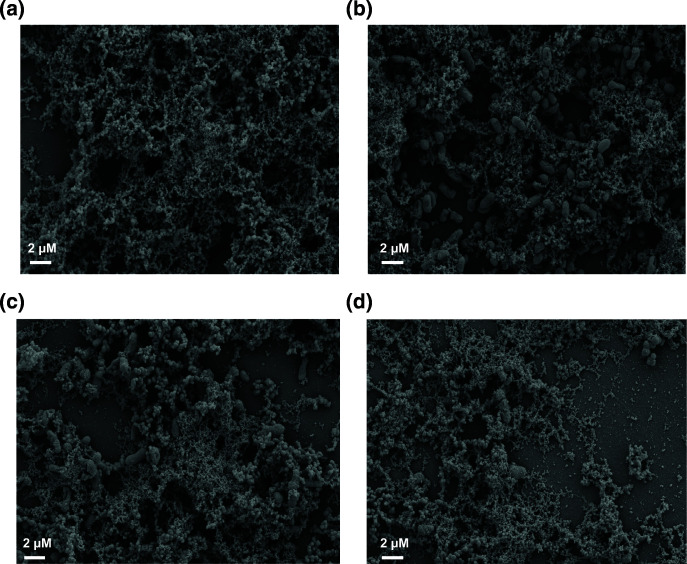
SEM of *K. pneumoniae* isolates that produce varying capsule polysaccharides. The figure shows hypermucoviscous KPPR1S (K2 isolate) (Fig. 6a) and a non-hypermucoviscous *K. pneumoniae* MKP103 (Fig. 6c) next to their respective capsule mutants (Fig. 6b, d). All figures show SEM images with 4000x magnification after fixation with the improved method.

We next investigated the ability of our fixation method to preserve biofilms from different *K. pneumoniae* isolates. We imaged biofilm formation of a K2 serotype hypermucoviscous isolate KPPR1S, a non-hypermucoviscous isolate MKP103, and their respective capsule mutants (*ΔwcaJ* and *Δwza*) [25, 26]. Overall, our improved fixation method captured robust biofilm structure with both strains and revealed strain and capsule dependent differences. More matrix material was associated with the hypermucoviscous isolate KPPR1S compared to the non-hypermucoviscous isolate MKP103 (Fig. 6a, c). KPPR1S (Fig. 6a) cells were surrounded by a denser matrix than MKP103 (Fig. 6c), with MKP103 cells left frequently visible. For both strains, there was greater biofilm formation by parental isolates (Fig. 6a, c) than their respective capsule mutants (Fig. 6b, d). This was particularly apparent with the hypermucoviscous isolate where loss of capsule (Fig. 6b) led to exposure of bacterial cells. Interestingly, although the capsular mutant biofilms were less dense (Fig. 6b, d) than their parental isolates (Fig. 6a, c) these isolates still have a considerable amount of matrix material surrounding the cells. These results indicate that our improved fixation method can capture nuances of biofilm structure for many *K. pneumoniae* strains.

## Discussion

In this study, we investigated parameters influencing the fixation of *K. pneumoniae* biofilm for SEM imaging. With the heterogenous nature of the biofilm structure, a complex primary fixative is necessary [13]. Furthermore, a standardized method would benefit the field as comparisons between different studies are more intuitive. We aimed to increase the preservation of the non-covalent interactions with a more complex fixative to prevent damage from the drying steps of SEM preparation that can affect delicate structures.

We found that a combination aldehyde fixative was beneficial for preserving biofilm. Extracellular biofilm matrix is composed of a number of biomolecules that interact using non-covalent interactions to maintain its structure [19]. The base for most SEM fixation steps includes glutaraldehyde to preserve structure by crosslinking the proteins the structure [30, 31]. Erlandsen *et al*. found that the combination of formalin and glutaraldehyde without a cationic dye was not able to preserve the exopolysaccharides of *K. pneumoniae* [23]. We also found that glutaraldehyde alone with a cationic dye was not sufficient to preserve the biofilm and that the combination aldehyde fixative is necessary. Furthermore, we found that tannic acid addition to this combination further increases the preservation of *K. pneumoniae* biofilm matrix material. This has shown promise for preserving Gram-positive biofilms of *Sc. wiggsiae* and *St. mutans* when added during a post-fixation step [6]. Tannic acid increased the biofilm preservation significantly and should be considered when performing SEM with *K. pneumoniae* biofilms.

In general, we found that ruthenium red was able to better preserve the cell-associated matrix, although alcian blue preserved surface associated material not preserved with ruthenium red. The greater preservation of these surface associated polysaccharide connections was also observed in *Enterococcus faecalis* [23]. Furthermore, we found the most substantial increase in biofilm preservation was provided by tannic acid. There is significantly more matrix material associated with the biofilms with tannic acid (Fig. 3) compared to those without (Fig. 2). Interestingly, we found that for *K. pneumoniae* biofilms the addition of tannic acid to the fixative was optimal because the addition after fixation led to loss of biofilm or artifact introduction. Therefore, caution should be used when using tannic acid after the primary fixative.

Our improved fixation method captured the biofilm architecture of three *K. pneumoniae* strains, suggesting it could be applicable for this bacterium. Comparing different *K. pneumoniae* isolates revealed distinct differences in biofilm formation and the impact of capsule. Our findings are in line with studies that found capsule mutants forming less robust biofilms [7, 35–37] but also highlights that the amount of matrix produced by these isolates may be sufficient to allow protection from the host immune system and antibiotic therapy. For example, Ernst *et al*. reported that although hypermucovisous isolates are more often the causative agent of pneumonias, capsule deficient isolates are often isolated from clinical urinary tract infections [38].

In conclusion, we have shown that the fixation step of SEM preparation strongly influences the observed biofilm structure, and the benefits of improving fixation for studying biofilms for comparative studies. While the method we describe here is an improvement over our basic method, a future focus of our work will be to test additional additives or changes in fixation parameters that may further enhance our ability to retain detailed features of natural biofilm structure and to perform quantification to reveal the changes in specific components of the biofilm with improved SEM fixation.

## Peer review history

### VERSION 2

#### Editor recommendation and comments

https://doi.org/10.1099/acmi.0.000470.v2.3

© 2023 Efthimiou G. This is an open access peer review report distributed under the terms of the Creative Commons Attribution License.

**Georgios Efthimiou**; University of Hull, Biomedical Science, Hardy Building, Cottingham Road, UNITED KINGDOM, Hull

Date report received: 02 January 2023

Recommendation: Accept

**Comments**: The work presented is clear and the arguments well formed. This study would be a valuable contribution to the existing literature. This is a study that would be of interest to the field and community. All comments by the reviewers were satisfactorily addressed.

#### SciScore report

https://doi.org/10.1099/acmi.0.000470.v2.1

© 2023 The Authors. This is an open-access article report distributed under the terms of the Creative Commons License.

#### iThenticate report

https://doi.org/10.1099/acmi.0.000470.v2.2

© 2023 The Authors. This is an open-access article report distributed under the terms of the Creative Commons License.

#### Author response to reviewers to Version 1

We thank the Reviewers for their helpful comments. Our responses (in red) follow each point and we have amended the manuscript accordingly. This includes adding supplemental images and updated the main figures accordingly.

**Reviewers' comments and responses to custom questions:**

Please rate the manuscript for methodological rigour

Reviewer 2: Satisfactory

Please rate the quality of the presentation and structure of the manuscript

Reviewer 2: Good

To what extent are the conclusions supported by the data?

Reviewer 2: Partially support

Do you have any concerns of possible image manipulation, plagiarism or any other unethical practices?

Reviewer 2: No:

If this manuscript involves human and/or animal work, have the subjects been treated in an ethical manner and the authors complied with the appropriate guidelines?

Reviewer 2: Yes:

Reviewer 2 Comments to Author: Reviewer summary:

The Authors have presented a workflow towards a standardised method of fixation for observation of Klebsiella biofilms by electron microscopy (EM). The Authors note that there is no current standardised fixation method for biofilm observation using EM, and that their work addresses the knowledge gap and could go on to assist other researchers.

The Authors use an iterative process of altering the composition of their fixative solution to preserve the exopolysaccharide matrix (EPS) which is commonly removed by harsh chemical fixatives. Ultimately, this leads to non-representative imaging or misreporting of biofilm morphology/composition in EM studies.

The Authors' methodologies proceed logically, and each step is informed by the previous result. The manuscript is well written and clearly communicated. However, there is no indication as to whether the data they show is representative (i.e., no indication of replicates - biological and technical) - the Authors may consider adding these as supplementary data and including replicate numbers in the main text.

Moreover, I believe that if the Authors mean to present this as a fully optimised method of fixation, then the manuscript requires additional experiments to support their claims: quantitative analyses of the EM images, titration experiments for the components of their fixative, and biochemical assays to quantify the preservation of EPS resulting from their fixation method. If not, it should caveated that further optimisation may be required according to factors such as pH and fixative concentration.

Based on the above, I recommend this manuscript for Major Revisions prior to further review. I include some revision points for the Authors' attention below.

We appreciate the Reviewers comments and address the specific points below. It was our oversight not to discuss replicates. All experiments were performed in biological triplicate. The images are representative of the results. We have added this statement to the results section. We agree that the use of the term “optimized” is not appropriate. We feel a focus on the representative figures in the main manuscript will allow easier interpretation for the reader. We have more correctly referred to our results as testing different fixation methods and identifying an improved version compared to our starting conditions. We have carried this concept through our edits.

Major points:

The findings referring to Figure 1 and Lines 113-121:

The Authors state that 'Cells fixed with only glutaraldehyde had very little matrix material covering the cells and adhering to the background'. The cells in Figure 1A & B look objectively similar between the figure panels. The small clusters in Fig1A have approximately the same adhesive matrix levels as the larger microcolony shown in Fig1B per area. How can the Authors confirm that there is more or less EPS without any biochemical testing or image analysis? I see that Fig 1B has more cells present, but that does not equate necessarily to the retention of EPS by using PFA.

We agree with this point and have edited the results section to highlight that more cells appear to be retained by the methods used to generate Figure 1B, but that we cannot confirm changes in EPS with our qualitative data.

I suggest that the Authors conduct a simple experiment to quantify the levels of EPS between the two groups presented in Fig1A and Fig1B. Following fixation, the biofilm should be stained using fluorescent EPS dyes (e.g., WGA-conjugates), and each sample imaged under the same acquisition parameters using a widefield or, preferably, a confocal microscope. Comparative fluorescence intensity analysis should determine if there is more/less EPS per area when fixed with GAH alone or GAH + PFA.

We agree that confocal imaging would be useful to quantify the polysaccharide content for this.

However, the goal of this study was to investigate how fixation procedures affected biofilm structure visualization. In some instance this was very obvious. For example in Figure 3 where subsequent addition of tannic acid greatly increased biofilm preservation. We have added a caveat that quantification of polysaccharide would need to be done using an additional confocal staining. We have also changed the language to state that more cells are present, but they have approximately the same adhesive matrix level.

The Authors should not claim higher/lower EPS levels based on images where this could be subjective without additional quantification.

Moreover, relating to Figure 1, there are some cropping artefacts along the bottom and sides of the images. The Authors should resubmit this figure without the cropping artefacts... when zooming in on the high-res .EPS files, it appears to be because there is a diagonal rotation/crop on the images.

We have edited the manuscript remove statements our EPS abundance. We have adjusted the cropping to eliminate cropping artefacts and have resubmitted this figure without the artifacts.

Lines 193-212 referring to 'detailed analyses':

The manuscript contains no direct quantitative analyses, but does present some high-quality electronmicrographs as figures. Line 200 & 211 convey that 'detailed analysis' has been conducted on the data to report these findings - but the findings are based on subjective observations. The Authors should either adopt quantitative image analysis to back up their claims or should completely redraft this section while refraining from the use of 'analysis(es)'.

We have reworded the section to reflect that no direct quantitative analyses have been done to quantify and compare the levels of polysaccharide and matrix material. Any description of ‘detailed analysis’ has been removed. The language has been modified to reflect the qualitative information that is attainable from the imaging.

I do not disagree that, for example, Figure 6 shows marked changes in biofilm organisation under different fixation conditions. However, the observation is not quantitative or detailed. It is an objective interpretation of the image data, or a qualitative assessment. This is completely fine, but it should not be communicated as 'detailed analysis' unless the Authors modify the workflow to include quantitative computational or biochemical analysis of EPS levels. As above, statements such as in Line 204, '…considerably more biofilm matrix.' should be revoked unless measured.

We have redrafted the section describing Fig.6 to remove the description ‘detailed analysis’ and any discussion of quantity of biofilm matrix.

Ensuring this is truly the optimal method:

Chemical fixation is also an imperative stage of some optical microscopy methods, particularly in SMLM imaging applications like dSTORM. Optical microscopists have developed many universal and sample-specific fixation protocols based on, as is here, aldehyde (with or without PFA) fixation or alcohol dehydration. One notable finding from these studies has been that the pH and concentration of fixative components (particularly PFA) can have a drastic effect on specimen integrity. The Authors have not presented any data from titration experiments that show the integrity of the biofilm following fixation using different concentrations of individual fixatives. Moreover, the Authors do not provide any indication as to why 2% PFA was selected, versus 1%, 3%, 4%, or even 0.5% which are all common for microbiological specimen preparation.

Comments regarding ‘optimal method’ is well taken. We agree that percentage as well as quality/grade/pH of fixative can affect the prep and ultimate interpretation of the resulting images. The fix percentages were selected as a representative good practice for a wide range of samples. In addition, given that biofilm is very ‘tissue-like’ in nature, composed of multiple components with structural elements, we used the aldehyde fixative mix generally used on tissue—a modified Karnovsky’s type that we use frequently and have published with. We have mentioned this in the methods and referenced the Karnovsky method:

Abstracts of Papers Presented at the Fifth Annual Meeting: The American Society for Cell Biology. (1965). *The Journal of Cell Biology*, *27*(2), 1A-149A. http://www.jstor.org/stable/1604673

Also, the extended fixation time (roughly 16 hours) allows plenty of time for the primary fixative to work, and slight differences in concentration (2% vs 3%) are unlikely to impact the outcome, particularly when enhanced by additional fix methods. All reagents were EM grade and the cacodylate buffer used to make them is ph 7.4. The fixes were made with commercially available, EM grade glutaraldehyde and paraformaldehyde and it is not typically to check the pH of final fixes unless we made the aldehyde solution ourselves.

How can the Authors be sure that this method is indeed optimal when they have not determined if there is a concentration-dependent relationship or dependency on the pH of the fixative?

Overall we have edited the manuscript and removed “optimization” from our description. Instead we describe our observations and improvements that are made in retaining biofilm structure under the different condition.

Minor points:

Line 66 - 'Gram' should be capitalised in 'gram-positive'.

Edit completed

Line 66-67 - The Authors suggest that references 6 & 18 only provide methods for Gram-positives, however, ref 18 also presents methods for Mannheimia haemolytica, a Gram-negative.

Edit completed

Line 67/426 - A. Mukherjee is not listed as an author on this citation, please update the reference (https://journals.plos.org/plosone/article/file?id=10.1371/journal.pone.0233973&type=printable).

Edit completed

Line 80 - To avoid confusion, the Authors should consider changing the shorthand of 'S. wiggsiae and S. mutans' to 'Sc. wiggsiae and St. mutans' or 'Scar. wiggsiae and Strep. mutans'.

Edit completed

Lines 126-129 - The authors provide a succinct summary of the structure of AB and RR. The inclusion of the chemical structures as a figure panel in Figure 2 may add non-specialist readers.

Edit completed. We have added the chemical structures of ruthenium red and alcian blue to the manuscript supplemental information Figure S1

Line 129 - please check tenses throughout. 'hypothesize' should be past tense here.

We have checked the tenses throughout the manuscript

Line 132 - A comma is required between 'that' and 'although' to complete the parenthesis.

Edit completed.

Line 138 - 'aclar' should be in all capitals with a '®' symbol afterwards.

Edit completed.

Line 155-159 - The previous figure had RR in panel A and AB in panel B. Could the Authors please change the order of all figure panels to be consistent with RR and AB, since that is the key change in many figures? If so, please update the relevant figure notations throughout the manuscript.

Edit completed.

Line 198 - Typo; the word 'formation' is missing from '…increased capsule may be an impediment…'.

Edit completed.

Line 235 - Have the Authors explored a 'best of both worlds' approach by testing a combined method with RR and AB?

We have not tried combining the two dyes to achieve the best of both worlds but appreciate the reviewers suggestion and will consider this when continuing to optimize this method.

Methods Section - as this is a methods paper, I request that the Authors provide all reagent supplier details/CAT numbers to aid readers in replicating the method as closely as possible. Can the Authors please clarify that they are using EM-grade glutaraldehyde, as I understand this has a substantial effect specifically on fixation for EM-quality specimens.

All reagents purchased were EM grade and this is mentioned in the text on lines 819-820. We have also included the catalog numbers and supplier for the reagents used for the EM preparation.

Line 292 - the Authors mentioned that they quantified the polysaccharide purified for their imaging experiments, would the Authors consider including this as Supplementary Info?

We have removed the analysis of purified polysaccharide during the revisions to eliminate discussion of specific components of the biofilm matrix. Based on above comments we have stated the qualitative nature of this study and will pursue quantification studies in future work.

Line 294 - Typo; 'aclar' should be in all capitals with a '®' symbol afterwards.

Edit completed.

Lines 299, 301, 302 - 'tetroxide' should be included after 'osmium'.

Edit completed.

Lines 303 & 306 - 'Osmium' should be lowercase, in keeping with the rest of the Methods section.

Edit completed.

Line 316 - Typo; 'Queens's' should be 'Queen's'.

Edit completed.

Line 317 - Typo; there were no hypervirulent strains used, I believe this should be 'hypermucovisous'.

The hypervirulent strain is hypermucovisous. However, we have changed hypervirulent to hypermucoviscous in line with how we used the strain for this paper.

Table 1 - Can the Authors please provide the concentrations (%) for the graded alcohols and HMDS referenced in the table?

A footnote was added for the graded alcohols % and for the ratio of the HMDS ratio wash steps.

Please rate the manuscript for methodological rigour

Reviewer 3: Very good

Please rate the quality of the presentation and structure of the manuscript

Reviewer 3: Very good

To what extent are the conclusions supported by the data?

Reviewer 3: Strongly support

Do you have any concerns of possible image manipulation, plagiarism or any other unethical practices?

Reviewer 3: No:

If this manuscript involves human and/or animal work, have the subjects been treated in an ethical manner and the authors complied with the appropriate guidelines?

Reviewer 3: Yes:

Reviewer 3 Comments to Author: This paper was a really interesting read. It is becoming increasingly obvious that the biofilm architecture is so important and even more so with the capsular and hypervirulent variants of Klebsiella. I am surprised to see how these small adjustments to the fixation protocol make such a difference, the optimised protocol presented here is clearly effective for maintaining the biofilm matrix.

I would love to see this technique used with different isolates of Klebsiella, especially with new or alterative therapeutics.

Thank you for the suggestions for using the technique with different isolates. We have included in Figure 6 a hypermucoviscous and non-hypermucovisous isolates next to their respective capsule mutants. In addition, we have published this method being used with bac7 (1-35) in a manuscript published earlier this year in Microbiology Spectrum. (Ref#20: Fleeman RM, Davies BW. Polyproline Peptide Aggregation with Klebsiella pneumoniae Extracellular Polysaccharides Exposes Biofilm Associated Bacteria. Microbiology Spectrum. 2022;10(2). doi: 10.1128/spectrum.02027-21.)

I think it would inform a lot of research and reveal secrets of the biofilm lifestyle! Very important for this manuscript to be shared.

The paper was written in a bit of a different style from the traditional manuscript, but I enjoyed how detailed and reasoned the explanations were. Thought the way the chemistry information and previous microbiology/tissue sample microscopy was brought together really strengthens the manuscript. It is very well-evidenced and clearly explained.

A couple of very minor comments where I would like a little more detail;

Line 292 - was the polysaccharide precipitated onto the glass cover slip? I am unclear on what surface the isolated polysaccharides were imaged?

The polysaccharide was dried onto glass coverslip in an unprecipitated form. The droplet was dried in ambient air for a while, then placed in a chamber with desiccant (not an aggressive drying, just to remove any signs of obvious moisture). However, with changes based on reviewer comments we have removed analysis of purified polysaccharide.

Figure 4 - are images A and B at the same magnification? I don't know if I am just mistaking matrix for cells in image A - the exposed cells in image B seem to be bigger?

The original image for Fig4A was 3kx and Fig4B was 5kx. We chose these images as they showed the artifacts produced by staining with TA after staining with AB or RR.

With our new replicate images we have selected images both with magnification of 4kx and have mentioned in the figure legends the image magnification for each set of images.

Can you please confirm if these experiments were performed in biological replicates?

Yes, all experiments were performed in biological triplicate and the images are representative. We have added this statement to the methods.

### VERSION 1

#### Editor recommendation and comments

https://doi.org/10.1099/acmi.0.000470.v1.5

© 2022 Efthimiou G. This is an open access peer review report distributed under the terms of the Creative Commons Attribution License.

**Georgios Efthimiou**; University of Hull, Biomedical Science, Hardy Building, Cottingham Road, UNITED KINGDOM, Hull

Date report received: 14 September 2022

Recommendation: Major Revision

**Comments**: The work presented is clear and the arguments well formed. This study would be a valuable contribution to the existing literature. This is a study that would be of interest to the field and community. The reviewers have highlighted major concerns with the work presented. Please ensure that you address their comments.

#### Reviewer 2 recommendation and comments

https://doi.org/10.1099/acmi.0.000470.v1.3

© 2022 Townsend E. This is an open access peer review report distributed under the terms of the Creative Commons Attribution License.

**Eleanor Townsend**; University of Exeter Medical School, UNITED KINGDOM

https://orcid.org/0000-0002-1531-1747

Date report received: 12 September 2022

Recommendation: Minor Amendment

**Comments**: This paper was a really interesting read. It is becoming increasingly obvious that the biofilm architecture is so important and even more so with the capsular and hypervirulent variants of Klebsiella. I am surprised to see how these small adjustments to the fixation protocol make such a difference, the optimised protocol presented here is clearly effective for maintaining the biofilm matrix. I would love to see this technique used with different isolates of Klebsiella, especially with new or alterative therapeutics. I think it would inform a lot of research and reveal secrets of the biofilm lifestyle! Very important for this manuscript to be shared. The paper was written in a bit of a different style from the traditional manuscript, but I enjoyed how detailed and reasoned the explanations were. Thought the way the chemistry information and previous microbiology/tissue sample microscopy was brought together really strengthens the manuscript. It is very well-evidenced and clearly explained. A couple of very minor comments where I would like a little more detail; Line 292 - was the polysaccharide precipitated onto the glass cover slip? I am unclear on what surface the isolated polysaccharides were imaged? Figure 4 - are images A and B at the same magnification? I don't know if I am just mistaking matrix for cells in image A - the exposed cells in image B seem to be bigger? Can you please confirm if these experiments were performed in biological replicates?

*Please rate the manuscript for methodological rigour*

Very good

*Please rate the quality of the presentation and structure of the manuscript*

Very good

*To what extent are the conclusions supported by the data?*

Strongly support

*Do you have any concerns of possible image manipulation, plagiarism or any other unethical practices?*

No

*Is there a potential financial or other conflict of interest between yourself and the author(s)?*

No

*If this manuscript involves human and/or animal work, have the subjects been treated in an ethical manner and the authors complied with the appropriate guidelines?*

Yes

#### Reviewer 1 recommendation and comments

https://doi.org/10.1099/acmi.0.000470.v1.4

© 2022 Anonymous. This is an open access peer review report distributed under the terms of the Creative Commons Attribution License.

**Anonymous.**

Date report received: 01 September 2022

Recommendation: Major Revision

**Comments**: Reviewer summary: The Authors have presented a workflow towards a standardised method of fixation for observation of Klebsiella biofilms by electron microscopy (EM). The Authors note that there is no current standardised fixation method for biofilm observation using EM, and that their work addresses the knowledge gap and could go on to assist other researchers. The Authors use an iterative process of altering the composition of their fixative solution to preserve the exopolysaccharide matrix (EPS) which is commonly removed by harsh chemical fixatives. Ultimately, this leads to non-representative imaging or misreporting of biofilm morphology/composition in EM studies. The Authors' methodologies proceed logically, and each step is informed by the previous result. The manuscript is well written and clearly communicated. However, there is no indication as to whether the data they show is representative (i.e., no indication of replicates - biological and technical) - the Authors may consider adding these as supplementary data and including replicate numbers in the main text. Moreover, I believe that if the Authors mean to present this as a fully optimised method of fixation, then the manuscript requires additional experiments to support their claims: quantitative analyses of the EM images, titration experiments for the components of their fixative, and biochemical assays to quantify the preservation of EPS resulting from their fixation method. If not, it should caveated that further optimisation may be required according to factors such as pH and fixative concentration. Based on the above, I recommend this manuscript for Major Revisions prior to further review. I include some revision points for the Authors' attention below. Major points: The findings referring to Figure 1 and Lines 113-121: The Authors state that 'Cells fixed with only glutaraldehyde had very little matrix material covering the cells and adhering to the background'. The cells in Figure 1A & B look objectively similar between the figure panels. The small clusters in Fig1A have approximately the same adhesive matrix levels as the larger microcolony shown in Fig1B per area. How can the Authors confirm that there is more or less EPS without any biochemical testing or image analysis? I see that Fig 1B has more cells present, but that does not equate necessarily to the retention of EPS by using PFA. I suggest that the Authors conduct a simple experiment to quantify the levels of EPS between the two groups presented in Fig1A and Fig1B. Following fixation, the biofilm should be stained using fluorescent EPS dyes (e.g., WGA-conjugates), and each sample imaged under the same acquisition parameters using a widefield or, preferably, a confocal microscope. Comparative fluorescence intensity analysis should determine if there is more/less EPS per area when fixed with GAH alone or GAH + PFA. The Authors should not claim higher/lower EPS levels based on images where this could be subjective without additional quantification. Moreover, relating to Figure 1, there are some cropping artefacts along the bottom and sides of the images. The Authors should resubmit this figure without the cropping artefacts... when zooming in on the high-res .EPS files, it appears to be because there is a diagonal rotation/crop on the images. Lines 193-212 referring to 'detailed analyses': The manuscript contains no direct quantitative analyses, but does present some high-quality electronmicrographs as figures. Line 200 & 211 convey that 'detailed analysis' has been conducted on the data to report these findings - but the findings are based on subjective observations. The Authors should either adopt quantitative image analysis to back up their claims or should completely redraft this section while refraining from the use of 'analysis(es)'. I do not disagree that, for example, Figure 6 shows marked changes in biofilm organisation under different fixation conditions. However, the observation is not quantitative or detailed. It is an objective interpretation of the image data, or a qualitative assessment. This is completely fine, but it should not be communicated as 'detailed analysis' unless the Authors modify the workflow to include quantitative computational or biochemical analysis of EPS levels. As above, statements such as in Line 204, '…considerably more biofilm matrix.' should be revoked unless measured. Ensuring this is truly the optimal method: Chemical fixation is also an imperative stage of some optical microscopy methods, particularly in SMLM imaging applications like dSTORM. Optical microscopists have developed many universal and sample-specific fixation protocols based on, as is here, aldehyde (with or without PFA) fixation or alcohol dehydration. One notable finding from these studies has been that the pH and concentration of fixative components (particularly PFA) can have a drastic effect on specimen integrity. The Authors have not presented any data from titration experiments that show the integrity of the biofilm following fixation using different concentrations of individual fixatives. Moreover, the Authors do not provide any indication as to why 2% PFA was selected, versus 1%, 3%, 4%, or even 0.5% which are all common for microbiological specimen preparation. How can the Authors be sure that this method is indeed optimal when they have not determined if there is a concentration-dependent relationship or dependency on the pH of the fixative? Minor points: Line 66 - 'Gram' should be capitalised in 'gram-positive'. Line 66-67 - The Authors suggest that references 6 & 18 only provide methods for Gram-positives, however, ref 18 also presents methods for Mannheimia haemolytica, a Gram-negative. Line 67/426 - A. Mukherjee is not listed as an author on this citation, please update the reference (https://journals.plos.org/plosone/article/file?id=10.1371/journal.pone.0233973&type=printable). Line 80 - To avoid confusion, the Authors should consider changing the shorthand of 'S. wiggsiae and S. mutans' to 'Sc. wiggsiae and St. mutans' or 'Scar. wiggsiae and Strep. mutans'. Lines 126-129 - The authors provide a succinct summary of the structure of AB and RR. The inclusion of the chemical structures as a figure panel in Figure 2 may add non-specialist readers. Line 129 - please check tenses throughout. 'hypothesize' should be past tense here. Line 132 - A comma is required between 'that' and 'although' to complete the parenthesis. Line 138 - 'aclar' should be in all capitals with a '®' symbol afterwards. Line 155-159 - The previous figure had RR in panel A and AB in panel B. Could the Authors please change the order of all figure panels to be consistent with RR and AB, since that is the key change in many figures? If so, please update the relevant figure notations throughout the manuscript. Line 198 - Typo; the word 'formation' is missing from '…increased capsule <formation> may be an impediment…'. Line 235 - Have the Authors explored a 'best of both worlds' approach by testing a combined method with RR and AB? Methods Section - as this is a methods paper, I request that the Authors provide all reagent supplier details/CAT numbers to aid readers in replicating the method as closely as possible. Can the Authors please clarify that they are using EM-grade glutaraldehyde, as I understand this has a substantial effect specifically on fixation for EM-quality specimens. Line 292 - the Authors mentioned that they quantified the polysaccharide purified for their imaging experiments, would the Authors consider including this as Supplementary Info? Line 294 - Typo; 'aclar' should be in all capitals with a '®' symbol afterwards. Lines 299, 301, 302 - 'tetroxide' should be included after 'osmium'. Lines 303 & 306 - 'Osmium' should be lowercase, in keeping with the rest of the Methods section. Line 316 - Typo; 'Queens's' should be 'Queen's'. Line 317 - Typo; there were no hypervirulent strains used, I believe this should be 'hypermucovisous'. Table 1 - Can the Authors please provide the concentrations (%) for the graded alcohols and HMDS referenced in the table?

*Please rate the manuscript for methodological rigour*

Satisfactory

*Please rate the quality of the presentation and structure of the manuscript*

Good

*To what extent are the conclusions supported by the data?*

Partially support

*Do you have any concerns of possible image manipulation, plagiarism or any other unethical practices?*

No

*Is there a potential financial or other conflict of interest between yourself and the author(s)?*

No

*If this manuscript involves human and/or animal work, have the subjects been treated in an ethical manner and the authors complied with the appropriate guidelines?*

Yes

#### SciScore report

https://doi.org/10.1099/acmi.0.000470.v1.1

© 2022 The Authors. This is an open-access article report distributed under the terms of the Creative Commons License.

#### iThenticate report

https://doi.org/10.1099/acmi.0.000470.v1.2

© 2022 The Authors. This is an open-access article report distributed under the terms of the Creative Commons License.

## Supplementary Data

Supplementary material 1
