# Supplementary material for: Investigating Klebsiella pneumoniae biofilm preservation for scanning electron microscopy
Source: Access Microbiol. 2023 Feb 3;5(2):000470.v3. doi: 10.1099/acmi.0.000470.v3 (PMC9996182; doi:10.1099/acmi.0.000470.v3)

Investigating *Klebsiella pneumoniae* Biofilm Preservation for Scanning Electron Microscopy.

Renee M. Fleeman[a*,#], Michelle Mikesh[b], Bryan W. Davies[a,c,d,*]

[a]Department of Molecular Biosciences, The University of Texas at Austin, Austin, TX 78712, USA

[b]Center for Biomedical Research Support, The University of Texas at Austin, Austin, TX 78712, USA

[c]Center for Systems and Synthetic Biology, The University of Texas at Austin, Austin, TX 78712, USA

[d]John Ring LaMontagne Center for Infectious Diseases, The University of Texas at Austin, Austin, TX 78712, USA

*Correspondence: renee.fleeman@ucf.edu, bwdavies@utexas.edu

ORCID account = https://orcid.org/0000-0002-2046-0341, https://orcid.org/0000-0001-7103-461X

[#]Current location: Division of Immunity and Pathogenesis, Burnett School of Biomedical Sciences, College of Medicine, University of Central Florida. Orlando, FL 32837.

Supplemental Information:

Supplemental Figures:

Figure S1.

Chemical structures of ruthenium red and alcian blue. Shown are the chemical structures for the cationic dyes used in this study. **Figure S1a** is ruthenium red and **Figure S1b** is alcian blue. The structures were created using Marvin Sketch 20.8.

A

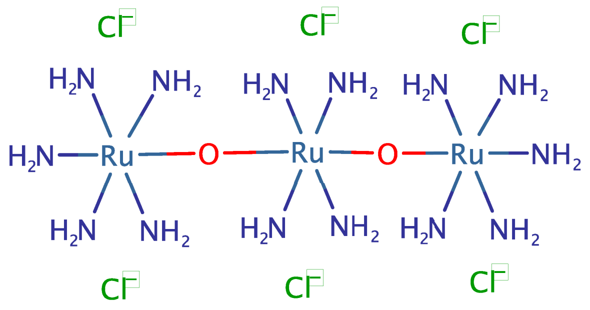

B

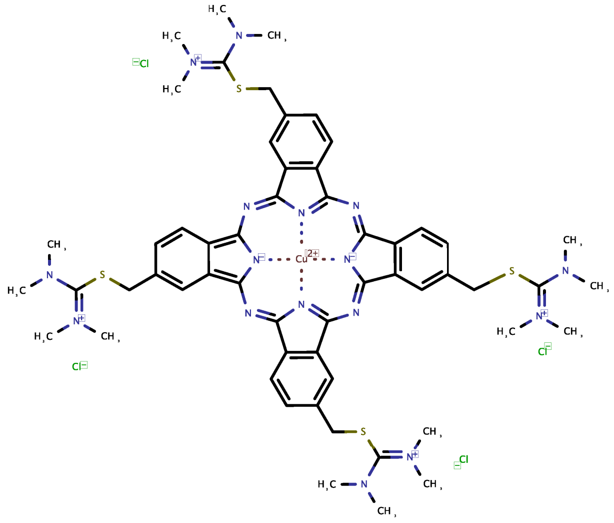

Supplement: Supplementary material 1 [file acmi-5-470.v3-s001.pdf]
